# Different Aspects of Kidney Function in Well-Controlled Congenital Hypothyroidism

**DOI:** 10.4274/Jcrpe.811

**Published:** 2012-12-19

**Authors:** Alaleh Gheissari, Mahin Hashemipour, Pooya Khosravi, Atoosa Adibi

**Affiliations:** 1 IUMS, Pediatric Nephrology, Isfahan, Iran, Islamic Republic of; 2 Isfahan University of Medical Sciences, Pediatric Endocrinology, Isfahan, Iran, Islamic Republic of; 3 Isfahan University of Medical Sciences, Pediatrics, Isfahan, Iran, Islamic Republic of; 4 Isfahan University of Medical Sciences, Radiology , Isfahan, Iran, Islamic Republic of

**Keywords:** congenital hypothyroidism, kidney function, kidney size

## Abstract

**Objective:** Congenital hypothyroidism (CH) increases the prevalence of kidney and urogenital malformations. There are limited studies considering different aspects of kidney function in well-controlled CH patients. We evaluated some features of kidney function in euthyroid children with CH who have been receiving thyroxine hormone since early life.

**Methods:** This cross-sectional study was conducted in Isfahan, Iran, on 74 children aged 2-15 years old (36 CH patients and 38 healthy children). Inclusion criteria for CH patients were euthyroidism at the time of the survey and initiation of replacement therapy during the early neonatal period. Kidney ultrasound evaluation was performed in all participants. Serum biochemistry included urea, creatinine, sodium (Na), potassium (K), magnesium, calcium, and cystatin C levels. Urine electrolytes, fraction excretion (FE) of electrolytes and microalbumin, and glomerular filtration rate (GFR) were also determined.

**Results: **The male/female ratio was 0.8/1 and 1.5/1 in the patient and control groups, respectively. Mean age and height did not differ significantly between the two groups. Ultrasound evaluation of the kidney revealed that the anteroposterior diameter of the right kidney was significantly higher in CH patients as compared to healthy subjects. No significant difference was observed between GFRs in patients with CH and healthy children. The mean values for FENa and FEK were significantly higher in the patient group.

**Conclusions:** Increased FENa and FEK may be a manifestation of impaired tubular maturation in CH. More longitudinal studies are needed to evaluate kidney function in CH patients.

**Conflict of interest:**None declared.

## INTRODUCTION

Thyroid hormones are of prime importance in human growth and development. Congenital thyroxine (T4) deficiency or insufficiency in the newborn results in severe mental retardation (1,2,3). Reported prevalence of congenital hypothyroidism (CH) varies between 1/748 and 1/10 000. These figures differ by geographic area and applied testing strategies (4,5,6,7).

The incidence of several congenital malformations including cardiac, oral, neurologic, and urogenital anomalies have been reported to be higher in CH patients in comparison with a normal population (8,9,10). CH increases the prevalence of renal and urogenital malformations (odds ratio: 13.2) (8). In addition, a delay in the treatment of CH or incomplete T4 replacement therapy in these patients may affect kidney size (1). The role of thyroid hormones on kidney tubular function and on the renin-angiotensin-aldosterone system has been studied by several groups (11,12). However, the effect of T4 replacement therapy shortly after birth on preservation of kidney function has not been investigated in depth. A neonatal thyroid screening program is being conducted in Isfahan since 2002. Following the initiation of this program, a notable decrease has been observed in the incidence of undiagnosed and uncontrolled CH cases. On the other hand, studies investigating the different aspects of kidney function in well-controlled CH patients are limited. In the present study, we evaluated different features of kidney function in euthyroid children born with CH and treated with T4 hormone started shortly after birth. 

## METHODS

This cross-sectional study on 74 children aged 2-15 years was conducted in Isfahan, Iran, from March 2010 to October 2011. Thirty-six subjects were known cases of CH who met the inclusion criteria of being euthyroid at the time of the survey and having received replacement therapy initiated in the early neonatal period (within the first 14 days of life). Thirty eight age-, weight- and height-matched healthy children were selected as the control group. Information on this group was based on their medical records and interviews conducted with their parents. Due to the scarcity of poorly controlled CH patients in the community, we were not able to recruit such patients to the study as another control group.

Kidney ultrasound was performed in each participant by the same experienced radiologist blinded to the diagnosis of CH using an ultrasound apparatus with 3 and 6 MHz curvy linear probes (Voluson 730 Expert, GE Medical Systems, Milwaukee, WI, USA). All three dimensions of each kidney (length, width, and height), as well as the anterior-posterior (AP) diameter of the pelvis, renal parenchymal thickness, and bladder wall thickness were measured. An AP diameter greater than 3 mm and a bladder wall thickness greater than 2 mm were accepted as high for all ages. Fasting blood samples were collected in all subjects to measure cystatin C, blood urea nitrogen (BUN), creatinine (Cr), magnesium (Mg), sodium (Na), potassium (K), and calcium (Ca). Urine Cr, Mg, Na, K, Ca, and microalbumin (MA) were measured in the fasting spot urine samples. MA was assessed by the nephelometric (turbidimetric) method, using a locally manufactured kit (Pars-Azmoon, kit number 2055015, Iran). A urine MA/Cr ratio greater than 30 was considered abnormally high. Serum Cr, Mg, Ca, and BUN were measured enzymatically on a Hitachi 7350 autoanalyzer. Serum cystatin C was measured by a particle-enhanced immunoturbidimetric method (Dako, Denmark). Fractional excretion (FE) of Na and K (FENa and FEK) were used to detect presence of renal tubular dysfunction. Additionally FEMg was used as a marker of tubular injury (13,14,15). We used the following formulae to calculate FENa, FEK, and FEMg (13):

FE(X) = [serum Cr ^X^ urinary (X)/serum (X) ^X^ urinary Cr] ^X^100

FEMg= [serum Cr ^X^ urinary (Mg)/serum (Mg) ^X^ urinary

Cr ^X^ 0.7] ^X^ 100

It has been shown previously that FEMg measurements can be used to demonstrate any possible damage in the thick ascending limb of Henle’s loop which is one of the most energy-dependent sections of the renal tubule (14,15).

Glomerular filtration rate (GFR) was measured using the following formulae:

1) GFR (Schwartz formula) = K ^X^ height (cm)/serum Cr

(mg/dL) (16)

(K= 0.55 for girls and K= 0.7 for boys)

2) GFR (mL/min/1.73 m^2^) (cystatin C-based Schwartzformula) = 39.1[height (m)/SCr(mg/dL)]^0.516^ [1.8/cystatinC (mg/L)]^0.294^ [30/BUN(mg/dL)]^0.169^ [1.099(male)][height (m)/1.4]^0.188^ (16).

3) GFR (cystatin C-based formula) = 84.69 ^X^ cystatin C(mg/L) −1.680 ^X^ 1.384 (17)

4) Log GFR (cystatin C-based formula) =1.962+1.123 _ log (1/cystatin C) (18)

Using the above equations enabled us to compare the GFR results by sex, height, and age.

Euthyroid state was defined as a normal response to thyroid-stimulating hormone (TSH) and normal T4 levels. In all CH patients included in the study, serum TSH values were kept at levels less than 5 mU/L and serum total T4 and free T4 during the first year of life within targeted values (130-206 nmol/L or 10-16 μg/dL and 18-30 pmol/L or 1.4- 2.3 ng/dL, respectively) (19).

The study protocol was approved by the Research Ethics Committee at Isfahan University of Medical Sciences. Written consent was obtained from each subject’s parents or caregivers, and also oral consent was taken from the participants, when appropriate.

## RESULTS

There were 20 females and 16 males in the study group (male/female ratio 0.8/1). There were 23 males and 15 females in the control group (male/female ratio 1.5/1). The mean age did not differ significantly between the study and control groups (9.2 ± 3.6 vs. 9.1 ± 4.1 years, respectively; p>0.05). Mean ± standard deviation (SD) values for height in the study and control groups were 132.9 ± 21.7 cm and 131.0 ± 20.8 cm, respectively (p>0.05). Height SD scores were also similar in the study and control groups (0.00 ± 1.00 vs. 0.00 ± 0.00, respectively; p<0.05). The median age at diagnosis and treatment in CH male and female patients were 10 and 14 days, respectively.

Kidney ultrasound results revealed that only AP diameter of the right kidney was significantly increased in CH patients as compared to healthy subjects (Table 1). Kidney echogenicity was normal in both groups. Bladder wall thickness was comparable in the two groups (1.05±0.23 mm in the patients vs. 1.05±0.22 mm in controls, p>0.05). A bladder wall thickness of more than 2 mm was observed in one patient with CH and in two subjects in the control group. A post-void residual urine volume of more than 10 mL was observed in one child with CH. A distal ureter diameter greater than 3 mm was also observed in one patient.

The mean levels of serum Na, K, Mg and Cr and the mean levels of urinary Na, K, Mg, and Cr were not significantly different between the study and control groups (data not shown). As shown in Table 2, the mean values for GFR, irrespective of the formula used for GFR calculation, were also comparable in patients and controls. In addition, the mean 1/cystatin C levels did not differ significantly between the two groups. The mean values for FENa and FEK were significantly higher in the study group (Table 2). No significant difference was observed in urinary Ca/Cr and urinary MA/Cr ratio between the two groups.

Since kidney measurements, serum Cr and GFR values all increase with age, we used SD scores to evaluate these parameters. Comparing SD scores for bladder wall thickness, serum Cr and GFR between the patient and control groups did not show any significant differences, except for the SD score of the kidney AP diameter being higher in the study group than in the control group (Table 3). 

## DISCUSSION

In this present study, we evaluated the anatomical and functional aspects of kidneys in well-treated CH children. Reports on evaluation of kidney function in uncontrolled CH are scarce and we were not able to compare our results in well-controlled CH cases with those on poorly controlled patients. Nonetheless, our results revealed kidney dysfunction in CH in addition to a bilateral mild increase in the AP diameter of the kidney in CH patients. However, no evidence of hydronephrosis (AP diameter of 10 mm or more) was found. Kumar et al (8) have reported a high incidence of congenital anomalies pertaining to the heart and to the gastrointestinal and urogenital systems in CH patients. They demonstrated that hydronephrosis was the most prevalent renal anomaly in CH children (8). In another study, an increased prevalence of urogenital malformations in CH was reported (9). Bulbul et al (1) have suggested that a delay in diagnosis and treatment could be the mechanism responsible for the change in kidney dimensions in CH children. Although a mild increase in AP diameter was detected also in our patients, overall geometric dimensions (length and width) of the kidneys in well-treated CH patients were found to be comparable to those of healthy children. Considering the significant correlation of kidney size with height in children (20), we recruited age- and height-matched healthy children as the control group in the present study.

In addition to the geometric parameters of the kidney, we also evaluated different aspects of kidney function, including GFR, urine MA , serum Cr levels, and FENa, FEK and FEMg. GFR assessment is one of the most important criteria for evaluation and detection of renal function impairment. Serum Cr-based Schwartz formula has been widely accepted to estimate GFR. Recently, a serum cystatin C-based equation has been developed to diminish the chance of overestimating GFR values measured by the Cr–based Schwartz formula (16). There are various equations to measure GFR based on serum cystatin C. We selected two equations that determined serum cystatin C with a similar technique as that used in our study (particle-enhanced immunoturbidimetric method) (17,18). In addition to GFR, various markers such as FENa and FEMg have been introduced to evaluate kidney function in the normal population and in chronic kidney disease (CKD) (14,15,21,22). Peralta et al (23) proposed a triple-marker approach using serum Cr, serum cystatin C, and microalbuminuria to screen CKD more accurately. We also added measurements of urine MA and FE of electrolytes to GFR to detect whether various aspects of kidney function differ in CH patients. The role of thyroid hormones on GFR has been widely discussed (24,25,26,27). It has been shown that serum Cr and cystatin C levels are affected by serum TSH in primary hypothyroidism and in CH (24,25,26,27). While Cr level is decreased in patients with primary hypothyroidism after T4 replacement therapy, it remains unchanged in central hypothyroidism after the same therapy (24). Asami et al (25) have shown that in infants with CH, the transient increase in serum Cr level disappears after T4 replacement. The possible mechanism suggested for decreased serum Cr after T4 replacement is that this treatment leads to increases in serum levels of vascular endothelial factor (VEGF) and insulin-like growth factor-1 (IGF-1). VEGF and IGF-1 are mediators that recover endothelial function and renal blood flow. Also these mediators may improve renal function in CH after T4 replacement (28). It has been indicated that high levels of serum Cr kinase, irrelevant to rhabdomyolysis, which were induced by severe hypothyroidism returned to normal values after replacement therapy (25).

Our results showed normal renal function in well-controlled CH patients. Using various equations to measure GFR, no difference was observed in GFR levels between CH patients and age- and height-matched healthy children in the present study. We could show that appropriate management of CH newborns maintains renal function in the normal range. Nonetheless, the mean values for FENa and FEK were found to be higher in our patients as compared with the control group.

It has been proposed that FENa can be used as a marker in assessing acute kidney injury (29,30). Decreasing K excretion and increasing Na excretion have been reported in CKD as well (31). Abnormal FENa and FEK values are reported to occur in different chronic diseases accompanied by CKD such as beta-thalassemia major, hydronephrosis, and renal artery stenosis, as well as in small for gestational age newborns (32,33,34,35). We demonstrated increased FENa and FEK but normal FEMg values and normal urine MA/Cr and urine Ca/Cr ratios in CH patients. The increased FENa and FEK levels in our patients demonstrate that some degree of renal tubular impairment is present in these patients. Mild impairment in tubular function may not be detected by determining GFR and even by testing for microalbuminuria. We measured urine MA using the turbidimetric method. Using more accurate methods may increase the chance of detecting microalbuminuria in the early stages of CKD. The role of T4 in kidney growth has been confirmed. Triiodothyronine (T3) is the main mediator in apoptotic and proliferative effects of the thyroid hormones at the cellular level (36,37). In addition, T3 is responsible for proximal tubule development (38). Whether replacing T3 changes the function and morphology of proximal tubular cells has not been studied. However, increased FENa and FEK may be a manifestation of impaired tubular maturation in CH. 

## Figures and Tables

**Table 1:**
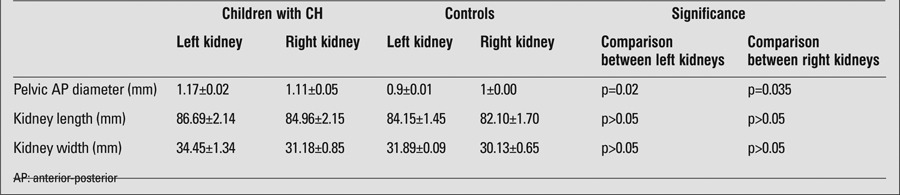
Kidney measurements in children with congenital hypothyroidism (CH) and in the control group (mean±SE)

**Table 2:**
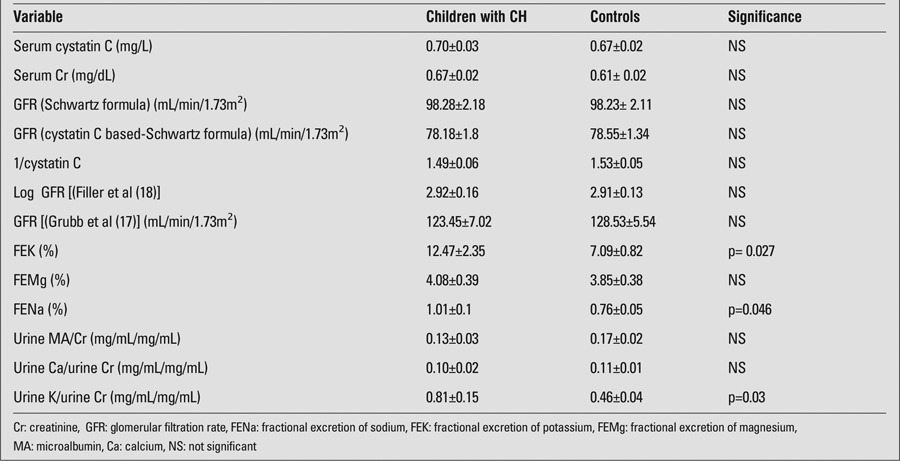
Markers of kidney function in children with congenital hypothyroidism (CH) and in the control group (mean±SE)

**Table 3:**
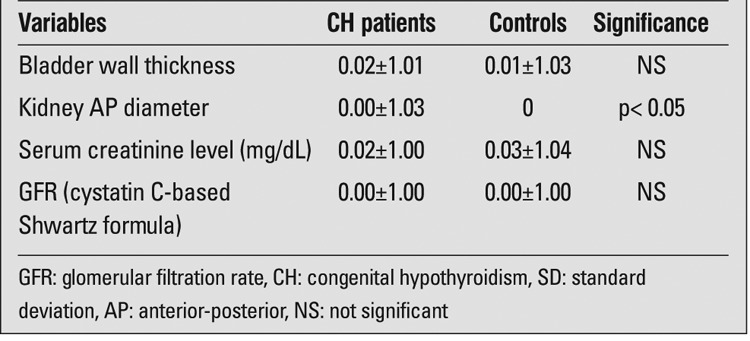
Markers of kidney function in children with congenital hypothyroidism (CH) and in the control group (mean±SE)
